# Remote ischemic preconditioning attenuates intestinal mucosal damage: insight from a rat model of ischemia–reperfusion injury

**DOI:** 10.1186/s12967-019-1885-4

**Published:** 2019-04-29

**Authors:** Lars Hummitzsch, Karina Zitta, Rouven Berndt, Yuk Lung Wong, Rene Rusch, Katharina Hess, Thilo Wedel, Matthias Gruenewald, Jochen Cremer, Markus Steinfath, Martin Albrecht

**Affiliations:** 1 Department of Anesthesiology and Intensive Care Medicine, University Hospital Schleswig-Holstein, Campus Kiel, Schwanenweg 21, 24105 Kiel, Germany; 2 Department of Cardiovascular Surgery, University Hospital of Schleswig-Holstein, Kiel, Germany; 3 Institute of Neuropathology, University Hospital Münster, Münster, Germany; 4 Institute of Anatomy, Christian-Albrechts-University, Kiel, Germany

**Keywords:** Hypoxia-inducible factor-1α, Intestinal ischemia, Ischemia/reperfusion injury, Matrix metalloproteinases, Remote ischemic preconditioning

## Abstract

**Background:**

Remote ischemic preconditioning (RIPC) is a phenomenon, whereby repeated, non-lethal episodes of ischemia to an organ or limb exert protection against ischemia–reperfusion (I/R) injury in distant organs. Despite intensive research, there is still an apparent lack of knowledge concerning the RIPC-mediated mechanisms, especially in the intestine. Aim of this study was to evaluate possible protective effects RIPC on intestinal I/R injury.

**Methods:**

Thirty rats were randomly assigned to four groups: I/R; I/R + RIPC; Sham; Sham + RIPC. Animals were anesthetized and the superior mesenteric artery was clamped for 30 min, followed by 60 min of reperfusion. RIPC-treated rats received 3 × 5 min of bilateral hindlimb I/R prior to surgery, sham groups obtained laparotomy without clamping. After I/R injury serum/tissue was analyzed for: Mucosal damage, Caspase-3/7 activity, expression of cell stress proteins, hydrogen peroxide (H_2_O_2_) and malondialdehyde (MDA) production, Hypoxia-inducible factor-1α (HIF-1α) protein expression and matrix metalloproteinase (MMP) activity.

**Results:**

Intestinal I/R resulted in increased mucosal injury (P < 0.001) and elevated Caspase-3/7 activity (P < 0.001). RIPC significantly reduced the histological signs of intestinal I/R injury (P < 0.01), but did not affect Caspase-3/7 activity. Proteome profiling suggested a RIPC-mediated regulation of several cell stress proteins after I/R injury: Cytochrome C (+ 157%); Cited-2 (− 39%), ADAMTS1 (+ 74%). Serum concentrations of H_2_O_2_ and MDA remained unchanged after RIPC, while the reduced intestinal injury was associated with increased HIF-1α levels. Measurements of MMP activities in serum and intestinal tissue revealed an attenuated gelatinase activity at 130 kDa within the serum samples (P < 0.001) after RIPC, while the activity of MMPs within the intestinal tissue was not affected by I/R injury or RIPC.

**Conclusions:**

RIPC ameliorates intestinal I/R injury in rats. The underlying mechanisms may involve HIF-1α protein expression and a decreased serum activity of a 130 kDa factor with gelatinase activity.

**Electronic supplementary material:**

The online version of this article (10.1186/s12967-019-1885-4) contains supplementary material, which is available to authorized users.

## Background

Remote ischemic preconditioning (RIPC) is a phenomenon, whereby repeated, non-lethal episodes of ischemia to an organ or limb exert protection against ischemia–reperfusion (I/R) injury in distant organs [1]. This effective, simple and low-risk procedure can be easily induced by transient occlusion of blood flow to an arm with a blood pressure cuff [2]. Despite intensive research there is still an apparent lack of knowledge about the RIPC-mediated mechanisms. Different studies indicated that besides neurogenic pathways and a systemic anti-inflammatory response, humoral mediators released into the systemic circulation by the RIPC-stimulus might play a key role in transferring the protective signal to the remote target tissues [1, 3].

Depending on the RIPC-mediated mechanisms, various physiological and pathophysiological processes can be influenced and regulated within the target tissue/organ. Several authors have shown that RIPC is able to attenuate apoptotic events induced by I/R injury in different organs [4–6]. Besides the regulation of apoptosis, preventing oxidation of cellular macromolecules by increasing antioxidant capacity within the target tissue seems to be an equally important mechanism of RIPC-mediated organ protection [7–9]. RIPC effects may also be mediated by hypoxia-inducible factor-1α (HIF-1α), a key transcription factor regulating cellular adaptation to hypoxia [10, 11], although the underlying mechanisms are only poorly understood so far [12, 13]. We have recently shown that RIPC is also able to reduce the activity of matrix metalloproteinases (MMPs) in serum and cardiac tissue of patients undergoing cardiac surgery and that these events are associated with decreased cellular injury in the target tissue, suggesting MMP activity as another potential factor in RIPC mediated organ protection [14, 15].

Experimental and clinical studies have mainly focused on the protective effects of RIPC on myocardial, cerebral and renal I/R injury. However, some studies also suggested beneficial effects of RIPC in the intestine [16, 17], in which I/R injury is associated with high mortality rates [18]. In the present study, we have established a rat model of intestinal I/R injury to evaluate possible protective effects of RIPC on intestinal I/R injury. Employing intestinal tissue and blood we furthermore analyzed the cellular and molecular mechanisms of intestinal I/R injury as well as the impact of RIPC on these events.

## Methods

### Animals and experimental setting

**Fig. 1:**
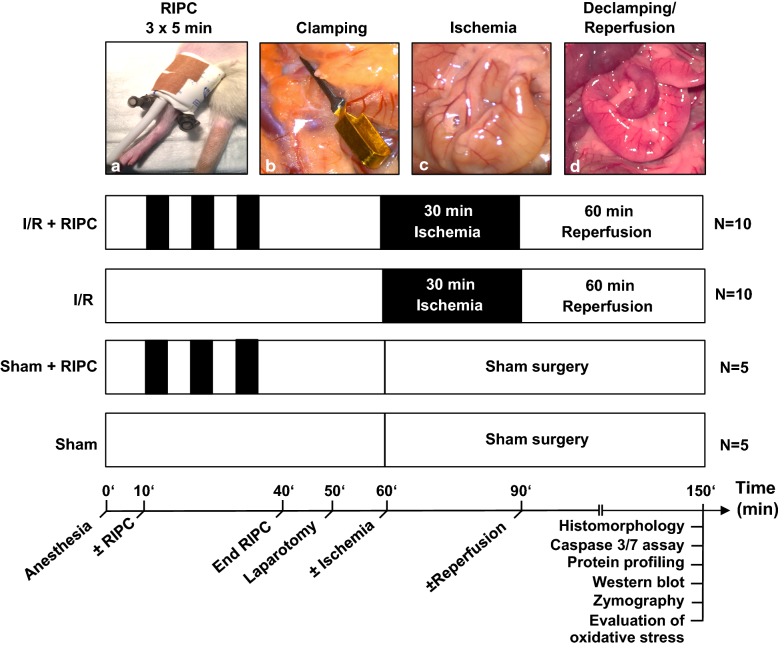
Experimental setting: rats were assigned to four groups: 1. intestinal I/R + RIPC (n = 10); 2. intestinal I/R (n = 10); 3. sham + RIPC (n = 5); 4. sham (n = 5). After induction of anesthesia and midline laparotomy, the superior mesenteric artery was clamped (**b**), followed by 30 min of intestinal ischemia (**c**) and 60 min of reperfusion (**d**). RIPC-treated rats received 3 × 5 min of bilateral hindlimb I/R (**a**) prior to surgery, while the sham-surgery groups received laparotomy without arterial clamping. At the end of the reperfusion period, serum and tissue samples were obtained and prepared for assessment of histomorphological injury, Caspase-3/7 activity, protein profiling, oxidative stress (malondialdehyde, hydrogen peroxide), western blotting and MMP activity (gelatin zymography)

Male Wistar rats (n = 30) weighing 366–480 g were obtained from the Animal Center of the University Clinic Schleswig-Holstein in Kiel. This study was approved by the Animal Care Committee of the local authority [Ministry of Energy, Agriculture, the Environment, Nature and Digitalization, protocol: V242-30460/2016(59-5/16)]. All procedures were performed according to the recommendations of the Guide for the Care and Use of Laboratory Animals of the National Institutes of Health. Animals had free access to food and water at any time before the experiments and were randomly allocated to four groups: (i) Intestinal I/R (n = 10), (ii) Intestinal I/R + RIPC (n = 10), (ii) Sham surgery (n = 5), (iv) Sham surgery + RIPC (n = 5). Anesthesia was induced by inhalation of 1–3% Sevoflurane and additional intraperitoneal injection of Ketamine (50 mg/kg). Adequate anesthesia during surgical intervention and the experiment was controlled based on the study protocol. Briefly, the following parameters were monitored/tested and anesthesia was—if necessary—adapted: (i) breathing frequency of the animal, (ii) interdigital reflex and (iii) defense reaction to defined surgical test stimuli (skin incisions). Midline laparotomy was performed and the superior mesenteric artery (SMA) was identified and isolated. The SMA was clamped by using a small atraumatic vascular clamp (Fig. 1b) and ischemia was verified by immediate lack of pulsation and discolouration of the small intestine (Fig. 1c). After 30 min of intestinal ischemia the vascular clamp was removed and the intestine was submitted to 60 min of reperfusion. Successful reperfusion was verified by an onset of pulsation and the recovery of the natural colour of the intestine (Fig. 1d). Sham operated animals received midline laparotomy without clamping the SMA. For induction of RIPC, a modified infant blood pressure cuff was placed around the upper third of each hindlimb and was inflated to 250 mmHg (Fig. 1a). RIPC-treated rats received 3 × 5 min of bilateral hindlimb I/R prior to surgery. After the reperfusion phase, animals were euthanized as part of a 2-step process, where anesthetized animals were exposed to increased concentrations of Sevoflurane (8%) and subsequently killed via exsanguination. 1 ml of blood for serum analyses was collected and 3 cm of jejunal tissue from similar anatomical regions was excised for protein analysis and quantification of intestinal mucosal damage.

### Evaluation of intestinal injury

For histomorphological analyses formalin (4%) fixed, paraffin-embedded intestinal samples were cut parallel to the gut axis into 5 µm thick sections and stained with hematoxylin and eosin. Samples were observed by light microscopy (Leica DMIL, Leica microsystems, Wetzlar, Germany) by two blinded investigators. The integrity of intestinal mucosa was graded by using the histological Chiu score [19]: Grade 0: normal mucosa; Grade 1: development of subepithelial Gruenhagen’s spaces at the tip of the villi; Grade 2: extension of subepithelial spaces with moderate lifting of the epithelial layer from the lamina propria; Grade 3: massive epithelial lifting down the side of the villi; Grad 4: completely denuded villi and dilated capillaries; Grade 5: loss of villi, disintegration of the lamina propria; hemorrhage and ulceration.

### Caspase-3/7 activity measurements

To evaluate the apoptotic activity in the intestinal tissue samples, the activity of the effector Caspases 3 and 7 were measured by using a rhodamine based fluorometric assay (Apo-One homogeneous Caspase-3/7 assay, Promega Corporation, Madison, USA). For evaluation of the Caspase activity, 10 µg of protein concentrate were treated and analysed on the basis of the manufacturer’s protocol using a fluorescence ELISA reader (Tecan, Crailsheim, Austria) in combination with the Magellan software v1.1.

### Cell stress protein profiling array

Protein profiling was performed using Cell Stress Protein Arrays (ARY018, R&D Systems, Minneapolis, USA) according to manufacturer`s protocol provided with the assay kit. Briefly, 200 μg of pooled intestinal protein samples (N = 5–10) were applied to the respective array membrane. Expression levels of 26 cell stress associated proteins were evaluated by densitometric analyses of the arrays using the ImageJ 1.41 software (National Institutes of Health NIH, Bethesda, USA). For each spot on the membrane, the optical density was measured and the cut off signal level was set to 10% of the respective reference spots. Only regulations of more than 20% were considered as relevant and were further analyzed.

### Hydrogen peroxide assay

Hydrogen peroxide concentrations were measured with a QuantiChrom™ Peroxide Assay Kit (Bio-Assay Systems, Hayward, USA). Briefly, 100 μl of detection reagent were added to 20 μl of serum and measurements were performed based on the manufacturer’s protocol. Samples were evaluated after 30 min in a 96-well plate at 585 nm using an ELISA reader (Tecan). Hydrogen peroxide concentrations in the samples were calculated from standard curves with known concentrations of hydrogen peroxide.

### Lipid peroxidation assay

To quantify oxidative stress, serum concentrations of malondialdehyde (MDA) were measured by using the Lipid Peroxidation (MDA) Assay Kit (ab118970; Abcam, Cambridge; UK) according to manufacturer’s protocol. Briefly, 20 µl of serum were incubated with 500 µl sulphuric acid and 125 µl phosphotungstic acid solution to precipitate lipids contained within the samples. After 5 min at room temperature and centrifugation at 13000×*g* for 3 min, the pellet was resuspended in 200 µl ddH_2_O containing 4 µl butylhydroxytoluol. Samples were then incubated at 95 °C for 60 min and subsequently cooled in an ice bath for 10 min. MDA was colourimetrically evaluated in a 96-well plate at 540 nm using an ELISA reader (Tecan). MDA concentrations were calculated from standard curves with known concentrations.

### Western blotting

Protein extraction was performed by homogenizing 200 mg intestinal tissue with 800 µl RIPA buffer containing 150 mM sodium chloride, 1% NP-40, 1% sodium deoxycholate, 0.1% sodium dodecyl sulphate (SDS) and 50 mM Tris–HCl (pH 7.6; all from Sigma-Aldrich, St. Louis, USA) with protease and phosphatase inhibitors (Roche, Basel, Switzerland). Afterwards, protein lysates were centrifuged for 10 min (21.000×*g*; 4 °C) and protein concentrations within the supernatants were determined with Roti^®^-Quant assays (Carl Roth, Karlsruhe, Germany). For western blotting experiments an equal amount of protein (50 μg) from each sample was mixed with 4× Laemmli buffer (8% SDS, 40% glycerol, 20% 2-mercaptoethanol, 0.008% bromphenol blue, 0.25 M Tris HCl, all from Sigma-Aldrich) and incubated for 3 min at 95 °C. Samples were separated by 8% SDS-PAGE and transferred to a PVDF membrane (Amersham Pharmacia Biotech, Piscataway, USA). After 2 h of blocking with TBST buffer containing 3% bovine serum albumin (Carl Roth) at room temperature, the membranes were incubated overnight at 4 °C with specific primary antibodies for HIF-1α (Novus Biologicals, Littleton, USA; 1:2.000) and β-actin (Santa Cruz, Heidelberg, Germany; 1:4.000). Signals from peroxidase-conjugated secondary antibodies (anti-rabbit, 1:16.000; Cell Signaling Technology, Danvers, USA and anti-goat, 1:25.000; Santa Cruz) were detected using the ECL kit (ECL-Plus Western blotting Detection Reagents, Amersham Pharmacia Biotech, Amersham, UK). Membranes were exposed to x-ray films and chemiluminescence intensities of the respective protein bands were analysed using the ImageJ software 1.41 (NIH). All western blots (HIF1-α, actin) were performed with the same samples and same membrane. The blots were first incubated with the HIF1-α/detection system and developed, then stripped using our previously described protocol [20] and reprobed with the actin/detection system as loading control.

### Gelatin zymography

Gelatin zymography was performed as described previously [15]. Briefly, 0.3 µl of serum or 2 µg of total protein concentrate were loaded and separated on 7% SDS polyacrylamide gels (containing 1 mg/ml gelatin) under non-reducing conditions. After electrophoresis, the gels were soaked in 2.5% Triton X-100 for 30 min to remove SDS and incubated in Tris–HCl (50 mmol/l, pH 7.5), containing CaCl_2_ (5 mmol/l) and ZnCl_2_ (1 mmol/l) overnight at 37 °C. After Coomassie blue staining, white bands of lysis indicated digestion of gelatin by MMPs. Densitometric analysis was performed using the ImageJ 1.41 software (NIH).

### Statistical analysis

Statistical analysis was performed with the software GraphPad Prism version 5.01 for Windows and data were tested for normality using the Kolmogorov–Smirnov test. If normality was not present, data were transformed (logarithm of x) before using parametric tests. Data were analysed by one-way analysis of variance and in cases of significant differences, adjusted for multiple comparisons (Tukey test). Variables are expressed as mean ± standard deviation (SD) and a P-value < 0.05 was considered to indicate a statistically significant difference.

## Results

### Effects of intestinal I/R injury and RIPC on mucosal injury and apoptosis

**Fig. 2:**
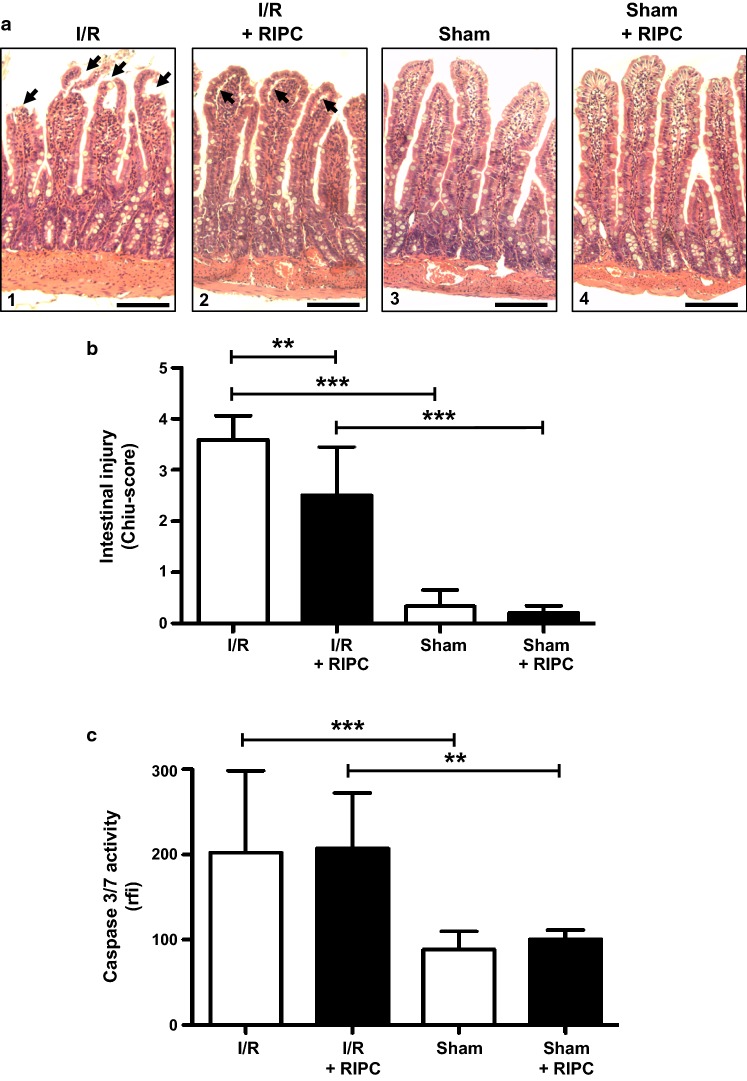
Intestinal injury: **a** representative photomicrographs of intestinal tissues stained with haematoxylin & eosin. **a1** Intestinal I/R: Severe mucosal injury with epithelial desquamation and denuded villi (arrows). **a2** Intestinal I/R + RIPC: RIPC before I/R injury results in minor mucosal injury represented by widened subepithelial spaces (arrows) and intact epithelial integrity at the villus tip. **a3**, **a4** Sham and Sham + RIPC: Both sham operated groups show an intact epithelial layer and villus structure. Scale bars represent 100 µm. **b** Scoring of intestinal damage. 30 min of ischemia followed by 60 min of reperfusion leads to a significant increase in Chiu-score. RIPC ameliorates the intestinal I/R injury and Chiu-score. **c** Enzymatic activity of Caspase 3 and 7 is significantly increased within the intestinal tissue after I/R injury, while RIPC does not influence Caspase activity. Columns show the mean of 5–10 independent experiments. Bars denote SD. **P < 0.01; ***P < 0.001. *rfi* relative fluorescent intensities

Intestinal I/R injury, induced by 30 min of ischemia followed by 60 min of reperfusion, resulted in clear signs of mucosal damage. Histomorphologically, predominantly bursted and denuded villi with epithelial desquamation were evident after I/R injury, while sham operated animals kept an intact intestinal epithelial layer and a normal villus structure. RIPC treated I/R animals exhibited an intestinal mucosa with less damaged villi and fewer epithelial desquamation, but more extended subepithelial spaces, mainly at the tip of the villi (Fig. 2a, 1–4). In addition, histomorphological scoring of intestinal I/R injury (Chiu-Score) revealed a significantly higher histological score in the intestine of I/R treated animals, indicating increased mucosal damage (Sham: 0.34 ± 0.32; Intestinal I/R: 3.59 ± 0.48; P < 0.001; Fig. 2b). Three cycles of bilateral hindlimb RIPC significantly reduced the Chiu-Score, suggesting tissue protective effects of RIPC (Intestinal I/R: 3.59 ± 0.48; Intestinal I/R + RIPC: 2.51 ± 0.94; P < 0.01; Fig. 2b). Besides histological signs of mucosal injury, I/R also lead to an increased activity of the pro-apoptotic Caspases 3 and 7 within the intestinal tissues (Sham: 88.51 ± 21.18 a.u.; Intestinal I/R: 202.10 ± 96.40 a.u.; P < 0.001; Fig. 2c). However, intestinal tissue activity of Caspase 3 and 7 was not influenced by RIPC (Intestinal I/R: 202.10 ± 96.40 a.u.; Intestinal I/R + RIPC: 207.10 ± 64.96 a.u.; P > 0.05; Fig. 2c). Additional analyses in which the activity/amount of LDH as a marker of cell damage was measured in sera of all animals showed a significant 24.7 ± 0.2% increase in LDH after I/R (compared to the sham group). RIPC reduced the levels of LDH to 10.5 ± 0.2% which was statistically not different from the respective sham group (Additional file 1: Figure S1).

### Effects of intestinal I/R injury and RIPC on cell stress protein expression

**Fig. 3:**
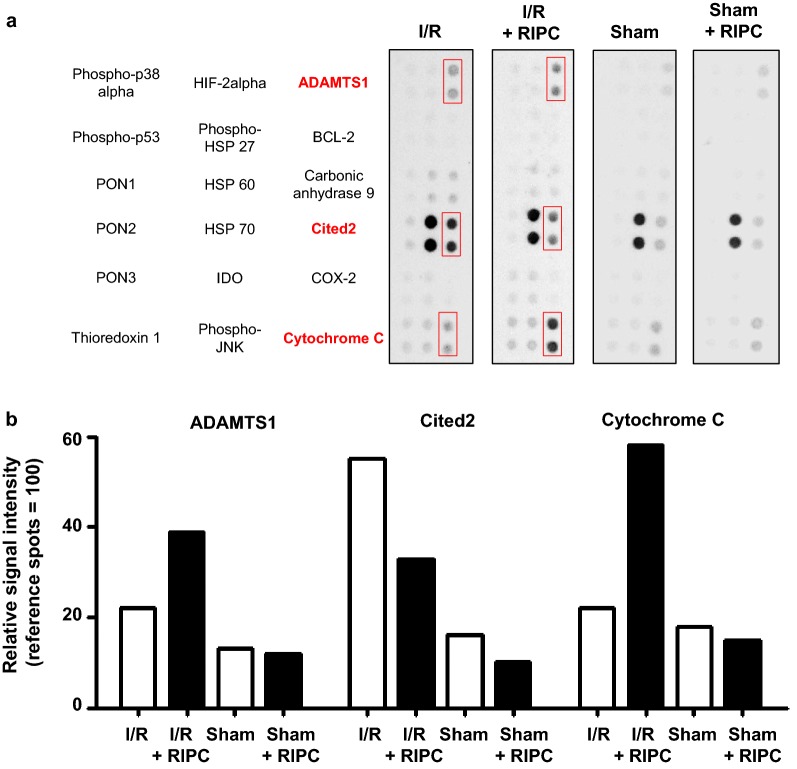
Cell stress protein array: Pooled samples of each study group were screened for expression of cell stress proteins. Each respective protein is represented by duplicate spots on the membrane. **a** Selected sections of the array membrane containing regulated proteins. Proteins indicated in red colour represent factors that are regulated by RIPC. **b** Evaluation of the relative protein expression of the regulated proteins ADAMTS1, Cited2 and Cytochrome C *a.u.* arbitrary units

Cell stress protein array analyses were performed with pooled samples of intestinal proteins to scan for possible factors involved in RIPC. The respective results showed an upregulation of a disintegrin and metalloproteinase with thrombospondin motifs 1 (ADAMTS1; Intestinal I/R vs. Sham: + 62%), cbp/P300 interacting transactivator with glu/asp rich carboxy-terminal domain 2 (Cited2; Intestinal I/R vs. Sham: + 232%) and Cytochrome C (Intestinal I/R vs. Sham: + 26%) after I/R-injury. RIPC affected the intestinal protein expression of ADAMTS1 (Intestinal I/R + RIPC vs. Intestinal I/R: + 74%), Cited2 (Intestinal I/R + RIPC vs. Intestinal I/R: − 39%) and Cytochrome C (Intestinal I/R + RIPC vs. Intestinal I/R: +157%) after the ischemic insult (Fig. 3).

### Effects of intestinal I/R injury and RIPC on hydrogen peroxide formation and lipid peroxidation

**Fig. 4:**
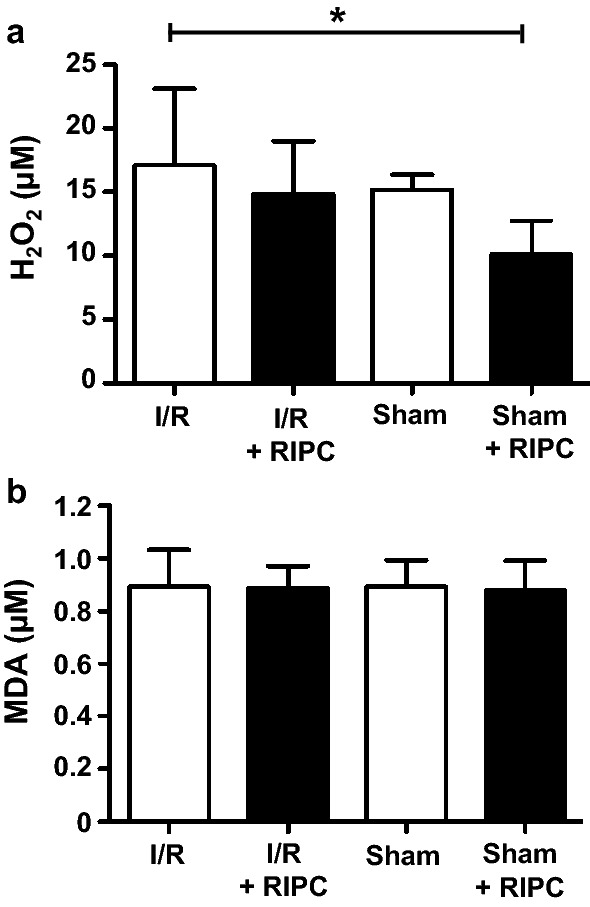
Hydrogen peroxide formation and lipid peroxidation: **a** Serum analyses after intestinal I/R injury reveals only a slight increase of H_2_O_2_ serum concentrations and there is a tendency of RIPC-mediated reduction of H_2_O_2_ serum concentration in the intestinal ischemia and sham-operated groups. **b** Serum concentrations of malondialdehyde (MDA) as a marker of lipid peroxidation are not changed after I/R injury or RIPC. Columns show the mean of 5–10 independent experiments. Bars denote SD. *P < 0.05

I/R induced organ damage is associated with oxidation of lipids, proteins and other molecules by reactive oxygen species (ROS) like hydrogen peroxide (H_2_O_2_). Malondialdehyde (MDA) is a product of lipid peroxidation and commonly used for quantification of oxidative stress [21]. Serum analyses revealed only a slight increase of H_2_O_2_ serum concentrations after intestinal I/R (Sham: 15.23 ± 1.15 µM; Intestinal I/R: 17.12 ± 6.02 µM; P > 0.05; Fig. 4a). There was a tendency of RIPC-mediated reduction of H_2_O_2_ serum concentration in the intestinal ischemia and sham-operated groups without reaching statistical significance (Sham: 15.23 ± 1.15 µM; Sham + RIPC: 10.11 ± 2.67 µM; Intestinal I/R: 17.12 ± 6.02 µM; Intestinal I/R + RIPC: 14.84 ± 4.19 µM; P > 0.05; Fig. 4a). MDA serum concentrations were not altered by I/R or RIPC (Sham: 0.89 ± 0.10 µM; Sham + RIPC: 0.88 ± 0.11 µM; Intestinal I/R: 0.89 ± 0.14 µM; Intestinal I/R + RIPC: 0.89 ± 0.08 µM; P > 0.05; Fig. 4b).

### Effects of intestinal I/R injury and RIPC on expression of HIF-1α

**Fig. 5:**
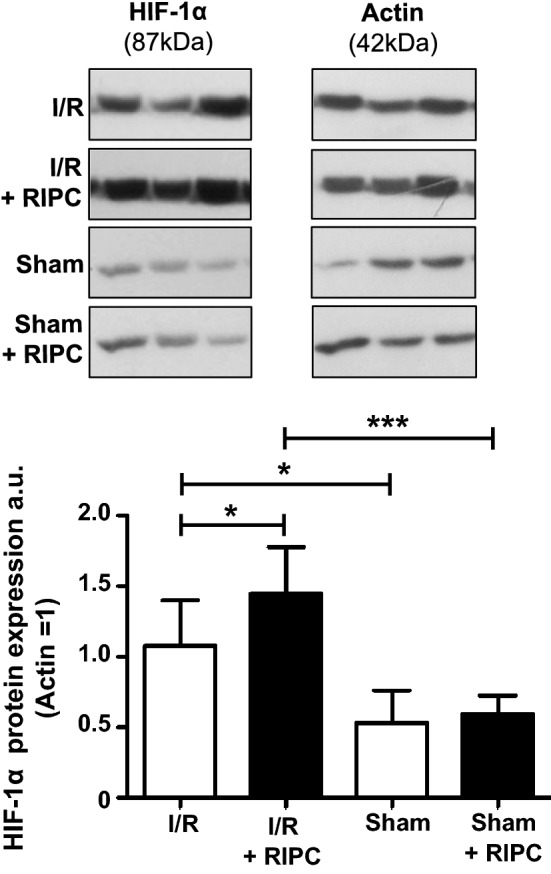
HIF-1α protein expression: Semiquantitative assessment of HIF-1α in unpooled/individual samples by western blotting shows an increased amount of HIF-1α after I/R-injury. RIPC significantly increases the amount of HIF-1α after I/R injury, but does not change the HIF-1 α protein levels in sham operated animals. Images on top show representative bands from 3 out of 5 or 10 experiments. Columns represent the mean of 5–10 independent experiments. Bars denote SD.*P < 0.05; ***P < 0.001. *a.u.* arbitrary units

HIF-1α is the key transcription factor regulating cellular adaptation to hypoxia and elevated HIF-1α levels were reported to be associated with intestinal protection under hypoxia [22]. Semiquantitative assessment of HIF-1α in unpooled/individual samples by western blotting showed an increased level of HIF-1α after I/R-injury (Sham: 0.53 ± 0.23 a.u.; Intestinal I/R: 1.07 ± 0.32 a.u.; P < 0.05; Fig. 5). RIPC significantly increased the amount of HIF-1α protein (Intestinal I/R: 1.07 ± 0.32 a.u.; Intestinal I/R + RIPC: 1.44 ± 0.33 a.u.; P < 0.05; Fig. 5), while in the sham operated animals RIPC-treatment did not increase the HIF-1α protein levels (Sham: 0.53 ± 0.23 a.u.; Sham + RIPC: 0.59 ± 0.13 a.u.; P > 0.05; Fig. 5). Additional proteome profiling arrays (Proteome Profiler Human Phospho-Kinase Array Kit, ARY003B, R&D Systems) detecting the phosphorylation status of 45 kinases were performed with pooled samples of all study groups. The results show an increased phosphorylation of 13/45 proteins in the I/R group (compared to the sham group). Interestingly, RIPC did not lead to an additional increase in phosphorylation of signaling kinases, instead 9 kinases even showed reduced phosphorylation status compared to the I/R group (Additional file 2: Figure S2).

### Effects of intestinal I/R injury and RIPC on matrix metalloproteinase activity

**Fig. 6:**
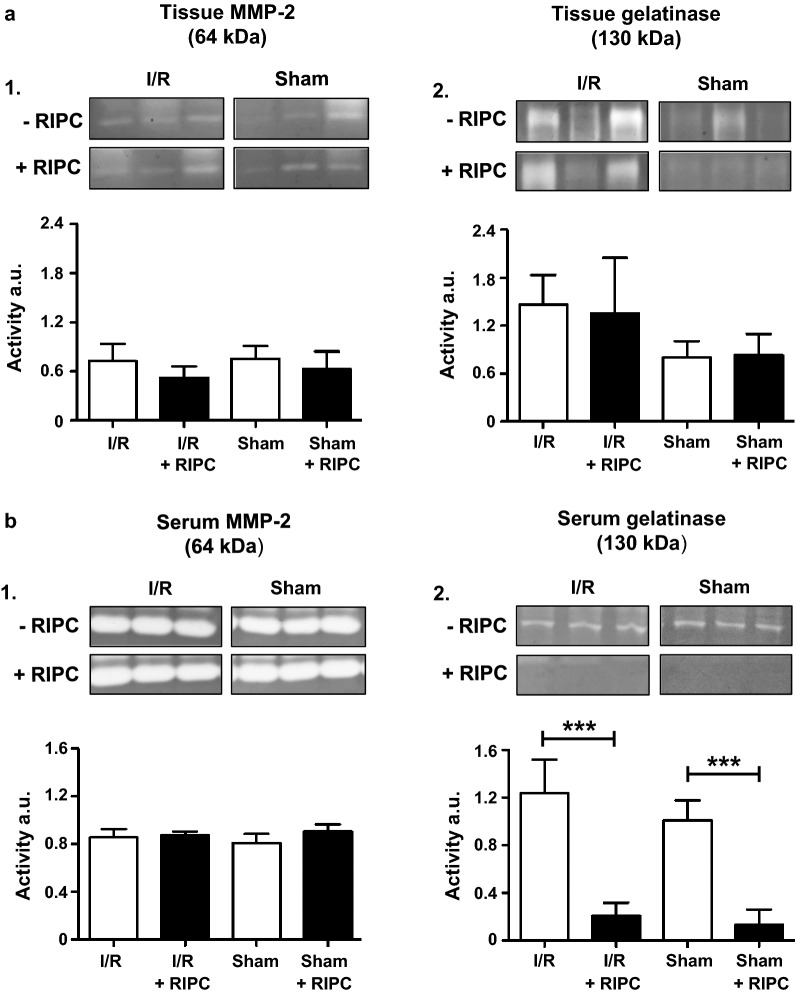
Activities of matrix metalloproteinases (MMP): **a** Tissue MMP activity: Neither I/R injury nor RIPC change the activity of MMP-2 in tissue samples (a1), whereas the gelatinase activity of a putative 130 kDa “Lipocalin/MMP-9” complex is by trend increased after I/R injury (a2). **b** Serum MMP activity: No differences in MMP-2 serum activity are detected between the groups (b1). However, intestinal I/R-injury results in a slightly increased serum gelatinase activity of the putative 130 kDa “Lipocalin/MMP-9” complex and RIPC significantly reduces the activity of this complex in all study groups (b2). Images show representative bands from 3 out of 5 or 10 experiments. Columns show the mean of 5–10 independent experiments. Bars denote SD. ***P < 0.001. *a.u.* arbitrary units

Activities of MMPs after intestinal I/R injury were evaluated in serum and intestinal tissue samples using gelatin zymography. Neither I/R injury nor RIPC changed the activity of tissue MMP-2 (Sham: 0.50 ± 0.10 a.u.; Sham + RIPC: 0.42 ± 0.14 a.u.; Intestinal I/R: 0.48 ± 0.14 a.u.; Intestinal I/R + RIPC: 0.35 ± 0.09 a.u.; Fig. 6a1). However, gelatinase activity at 130 kDa was by trend increased after I/R injury in intestinal tissues (Sham: 0.80 ± 0.21 a.u.; Intestinal I/R: 1.46 ± 0.37 a.u.; P > 0.05; Fig. 6a2). Although strong activity of MMP-2 was detected in serum, no significant differences were found between the groups (Sham: 0.81 ± 0.08 a.u.; Sham + RIPC: 0.91 ± 0.06 a.u.; Intestinal I/R: 0.86 ± 0.07 a.u.; Intestinal I/R + RIPC: 0.87 ± 0.03 a.u.; Fig. 6b1). However, intestinal I/R-injury resulted in a slightly increased serum gelatinase activity of the 130 kDa complex (Sham: 1.01 ± 0.07 a.u.; Intestinal I/R: 1.24 ± 0.09 a.u.; P > 0.05; Fig. 6b2), and RIPC was able to reduce the activity of this complex in all study groups (Sham: 1.01 ± 0.17 a.u.; Sham + RIPC: 0.13 ± 0.13 a.u; P < 0.001; Intestinal I/R: 1.24 ± 0.28 a.u.; Intestinal I/R + RIPC: 0.21 ± 0.11 a.u.; P < 0.001; Fig. 6b2).

## Discussion

Remote ischemic preconditioning (RIPC) can be simply induced by repeated cycles of transient occlusion of blood flow to a limb by inflating a blood pressure cuff and exerts potent protection from I/R injury in different organs. The majority of RIPC studies mainly focused on the potential effects of RIPC on myocardial, cerebral and renal I/R injury [23, 24]. Although intestinal I/R-injury is associated with extremely high mortality rates and RIPC could represent a promising treatment option, the RIPC-mediated effects and the underlying mechanism in the intestine are only poorly investigated.

Dickson et al. showed for the first time in a rat model that RIPC is able to increase the hypoxic tolerance within the intestine after I/R injury. Effluents from preconditioned hearts restored the intestinal motility after I/R-injury in the isolated rat jejunum, suggesting that humoral mediators are responsible for the protective effects [25]. Saeki et al. revealed, that 3 cycles of 15 min RIPC reduce the intestinal injury in a rat model of small bowel transplantation [26], while other studies using only one cycle of hindlimb ischemia did not show protective effects on intestinal I/R injury [27]. Based on the observation that repeated cycles of I/R are required for RIPC to be efficient, in the rat model employed in this study, repeated bilateral hindlimb RIPC (3 × 5 min) was applied directly before induction of intestinal I/R-injury (30 min of ischemia and 60 min of reperfusion). Our decision to use bilateral hindlimb ischemia instead of conditioning only one location originated from observations of animal studies that suggested that bilateral RIPC might be more effective than unilateral RIPC [28, 29].

Histomorphological analyses of the intestinal tissues revealed fewer signs of mucosal injury in RIPC treated animals compared to the control group (I/R injury only). Moreover, we showed a statistically significant reduction of the intestinal injury score (Chiu-Score) in the RIPC group, indicating cell and tissue protective effects of RIPC in our model. Our results are concordant with the findings from Saeki et al. implicating the existence of an “early window” of RIPC-mediated protection in the intestine [26], similar to the described windows for cardioprotection, where a biphasic course of RIPC-mediated protection with an early and late window was proposed [30]. In the heart, and possibly also in our intestinal I/R model, the early window of protection is believed to be caused by a fast activation of intracellular signalling pathways involving already preformed molecules, while the late window of organ protection is characterised by (slower) transcriptional changes and de-novo synthesis of protective proteins [30, 31]. LDH measurements in sera of all animals revealed a significant increase in LDH release after I/R. RIPC reduced the levels of LDH to values that were statistically not different from the sham group. Although serum LDH values of the I/R and IR + RIPC group did not differ significantly, the results are in line with the histological findings and Chiu-scoring. The lack of statistically significant differences between the I/R and I/R + RIPC group and the only moderate increase of LDH release after I/R might be due to the fact that blood and serum were obtained 60 min after the ischemic insult and that this time point might have been too late to detect a significant systemic increase of LDH activity. This hypothesis is supported by the results of others who have shown that levels of serum markers for intestinal cell damage are increased directly at reperfusion and not at later time points [32].

Several authors suggested that anesthesia is a confounder of organprotection by RIPC especially in the heart and that propofol anesthesia impairs the cardioprotective effect of RIPC [33, 34]. According to a meta-analysis by Zhou et al. and a recently published study by Cho et al. use of volatile anesthetics also attenuates the cardioprotection afforded by RIPC [35, 36]. Although, no studies have so far investigated whether or not anesthesia is also a confounder of intestinal protection by RIPC, we cannot rule out this possibility. In our study, in all animals and all treatment groups (including controls) interventions including the RIPC procedure were performed on the “background” of sevoflurane anesthesia and under these conditions RIPC was able induce protection from intestinal I/R injury. Therefore, RIPC is effective, even under sevoflurane anesthesia. Nevertheless, it remains unclear whether RIPC mediated intestinal protection is to some extent influenced by sevoflurane or if sevoflurane does alter secondary parameters of I/R and RIPC (e.g. protein expression, hydrogen peroxide generation, MMP activity).

Whereas intensive research over the last years has identified several proteins that are responsible for the RIPC-mediated mechanism of cardioprotection, there is an apparent lack of knowledge concerning the respective mediators in the intestine. To screen for possible RIPC-regulated proteins, antibody-based cell stress protein arrays were employed. These arrays simultaneously detect the expression levels of 26 proteins involved in cell stress and cellular defence mechanisms. Our results showed that compared to sham operated animals Cytochrome C, Cited2 and ADAMTS1 were strongly expressed in intestinal tissues from rats submitted to I/R. RIPC applied directly before the induction of I/R injury resulted in higher expression levels of Cytochrome C and ADAMTS1, while Cited2 was downregulated by RIPC.

Interestingly, Cytochrome C, a component of the mitochondrial electron transport chain, which plays also an important role in the initiation of apoptosis [37], was upregulated by RIPC. This observation is unexpected as we detected protective effects of RIPC on the intestinal I/R injury and would assume reduced apoptosis. It is commonly accepted that translocation of mitochondrial Cytochrome C into the cytosol leads to complex formation of Cytochrome C, Caspase-9 and Apaf-1, the so-called Apoptosome, which initiates cellular death by the activation of effector Caspases like Caspase 3 and 7 [37, 38]. Besides its role in electron transfer in the respiratory chain and the initiation of apoptosis, Cytochrome C is also known to possess antioxidative functions [37, 39]. Some studies reported an early increase of mitochondrial Cytochrome C protein levels after drug-induced apoptosis and these results were interpreted as an early cellular defence mechanism to avoid apoptosis [40–42]. In our experimental setting total protein of intestinal tissue samples was isolated and fractional analyses were not performed. Therefore, we cannot discriminate between effects caused by cytosolic or mitochondrial Cytochrome C and can only speculate about the role of the molecule in RIPC mediated mucosal protection. However, Caspase 3 and 7 activities in the intestinal tissues were significantly increased after I/R, suggesting that at least some of the histomorphologically visible mucosal injury is related to apoptosis. Interestingly, RIPC did not alter the Caspase activity in any of our study groups although RIPC was able to reduce mucosal injury. These findings suggest that RIPC does not reduce I/R injury via an attenuation of Caspase 3 and 7 mediated apoptosis and that the upregulation of Cytochrome C by RIPC in our animal model is most likely not related to apoptotic events via of Caspase 3 and 7. To further analyse possible antioxidative effects of RIPC, H_2_O_2_ formation and malondialdehyde (MDA) serum levels were measured. Neither intestinal I/R injury nor RIPC were able to change MDA serum levels. However, there was a slight increase of H_2_O_2_ serum concentrations after I/R injury and a trend of reduced H_2_O_2_ levels in all RIPC groups.

Proteome profiling revealed a RIPC-mediated increased expression of ADAMTS1 within the intestine after I/R injury. ADAMTS1 (a disintegrin and metalloproteinase with thrombospondin motifs 1) is a member of the MMP family involved in several physiological and pathophysiological processes like cellular differentiation, angiogenesis, inflammation, cancer development and arteriosclerosis [43]. Only little is known about the exact role of ADAMTS1 during hypoxia and I/R-injury. However, several authors suggested a HIF1-α induced upregulation of ADAMTS1 under hypoxia in endothelial cells and an early expression after myocardial infarction [44, 45]. Recently, studies from our group indicated an involvement of MMPs in RIPC-mediated organ protection [14, 15]. As ADAMTS1 also belongs to the class of MMPs, we extended our study and further evaluated the activities of different MMPs after I/R injury using gelatin zymography. Our results did not show any regulation of MMP-2 activity in intestinal tissue or serum, although several studies suggested that in heart, brain, kidney and liver a decreased MMP-2 activity after I/R injury is associated with reduced tissue injury and organ dysfunction [14, 46, 47]. Instead, intestinal I/R injury lead to a slightly increased gelatinase activity at 130 kDa in the serum samples and RIPC almost completely abolished this activity in the I/R and sham group. Other authors have described a gelatinase activity containing molecule of 130 kDa as Lipocalin-2/MMP-9 complex and contributed its function to inflammatory processes and tumour invasion [48]. Lipocalin-2 increases the proteolytic activity of MMP-9 and also protects the enzyme from degradation [48]. Whilst a reduced activity of MMP-9 is known to be associated with protection against I/R injury [49, 50], the possible effects of Lipocalin-2 and Lipocalin-2/MMP-9 complex on I/R injury is still not clear and controversially discussed [51–54].

As mentioned above, protein profiling of intestinal tissues revealed an increased expression of Cited2 after intestinal I/R injury, whereas RIPC was associated with decreased expression levels of Cited2. Cited2 is reported to be a negative regulator of HIF-1α and is activated under hypoxic conditions [55]. HIF-1α, a key transcription factor regulating cellular adaptation to hypoxia by activating several genes involved in antioxidative defence, apoptosis, glucose metabolism and angiogenesis, is promoting cellular survival during hypoxia [10, 31]. Whereas several studies indicated a pivotal role of HIF-1α in the process of local preconditioning, only little is known about the effects of HIF-1α during RIPC-mediated organ protection [12, 13]. Previous studies from our group demonstrated, that RIPC is able to increase HIF-1α protein levels in cardiac tissues from patients undergoing cardiac surgery and that this effect is associated with decreased Troponin-T levels as marker for cardiac injury [56]. Moreover, Weber et al. demonstrated that RIPC plasma protects human endothelial cells cultured in vitro from hypoxia-induced cell damage and that HIF-1α could play a key role in these events [20]. These results are also in accordance with newer studies demonstrating, that a RIPC-induced upregulation of HIF-1α is linked to decreased tissue injury after cerebral [57] and myocardial I/R [58, 59]. Our western blot analyses of intestinal tissues derived after I/R injury showed that RIPC leads to significantly increased HIF-1α protein levels, supporting the hypothesis, that RIPC is able to increase HIF-1α levels within the target tissue. Although current knowledge assigns HIF-1α a central role in RIPC mediated protection, further studies (i.e. using HIF-1α knockout models) are necessary to clearly show the direct involvement of HIF-1α in organ protection, especially in the intestine. Proteome profiling arrays detecting the phosphorylation status of 45 kinases showed an increased phosphorylation of 13/45 proteins in the I/R group (compared to the sham group). Interestingly, RIPC did not lead to an additional increase in phosphorylation of signaling kinases. These results are somewhat surprising, as numerous authors have postulated that the protective effects of RIPC in the heart are associated with an increased phosphorylation of pro-survival kinases (e.g. Erk1/2 and Akt) [12, 60, 61]. The increased phosphorylation of several signaling molecules by I/R may be interpreted as an intrinsic cellular defense mechanisms to control I/R-induced damage. Our observation, that RIPC does not further increase the phosphorylation status of these molecules suggests that the kinases investigated in our study are not responsible for the described protective effects of RIPC on intestinal I/R-injury and that RIPC-mediated protective mechanisms in the intestine differ from the mechanisms described for the heart. However, compared to the heart which is predominantly composed of cardiomyocytes, the composition of intestinal tissue is more complex, including various different cell types such as endothelial cells, epithelial cells, fibroblasts, immune cells etc. As the signaling arrays were performed using intestinal tissue homogenates, RIPC-mediated effects on one cell type (e.g. endothelial cells) might be masked by other cell types which did not respond to RIPC. We can also not exclude the possibility, that the time period from induction of RIPC (T 10 min) to sample preparation (T 150 min) might have been too long so that RIPC induced phosphorylation had already decreased.

There are several potential limitations of our study that need to be discussed: (i) As a result of the experimental setup, intestinal tissue samples could only be obtained at one time point at the end of the reperfusion period. Therefore, we cannot provide information about the temporal dynamics of I/R and RIPC associated cellular and molecular mechanisms. Regarding the ROS production, Caspase activity and LDH release, earlier time points may provide better insights into possible RIPC mediated antioxidative, antiapoptotic and cell protective effects, while later time points may be more suitable in revealing differences in MMP expression/activity. (ii) Human cell stress proteome profiling arrays were employed in the study. Although the manufacturer confirmed the suitability and cross reactivity of the arrays with rat tissue, we cannot exclude slightly different binding capacities of the array specific antibodies. However, arrays were performed with pooled tissue samples and only used as screening tool for the detection of cell stress proteins and pathways that could be involved in I/R-injury and RIPC. The respective proteins or downstream targets were in the following steps further analysed using unpooled samples and different biochemical methods. (iii) Although there is convincing evidence that the 130 kDa factor with gelatinase activity resembles a Lipocalin-2/MMP-9 complex, further biochemical analyses are necessary to confirm this hypothesis beyond doubt. (iv) Our study suggests that RIPC underlying mechanisms involve HIF-1α, ADAMTS1, Cited2, Cytochrome C and a decreased serum activity of a 130 kDa factor with gelatinase activity. However, functional interactions or a causal connection of these molecules were not demonstrated and further experiments (e.g. expression knock-down or function blocking studies) are needed to substantiate the findings of our work.

## Conclusions

Taken together, our results show RIPC mediated protection against intestinal I/R injury in rats and suggest that the underlying mechanisms may involve HIF-1α and a decreased serum activity of a 130 kDa factor with gelatinase activity. Further work has to clarify, whether RIPC also has the potential to preserve intestinal barrier function in patients suffering from intestinal I/R injury and is able to reduce the devastating consequences of intestinal I/R-injury in the clinic.

## Additional files

**Additional file 1.** Effects of I/R and RIPC on LDH release.

**Additional file 2.** Effects of I/R and RIPC on phosphorylation of cellular signaling kinases.

## Abbreviations

ADAMTS1: a disintegrin and metalloproteinase with thrombospondin motifs 1
HIF-1α: hypoxia-inducible factor-1α
H_2_O_2_: hydrogen peroxide
I/R: ischemia/reperfusion
MDA: malondialdehyde
MMP: matrix metalloproteinase
RIPC: remote ischemic preconditioning
ROS: reactive oxygen species
SMA: superior mesenteric artery
