# Supplementary material for: Remote ischemic preconditioning attenuates intestinal mucosal damage: insight from a rat model of ischemia–reperfusion injury
Source: J Transl Med. 2019 Apr 29;17:136. doi: 10.1186/s12967-019-1885-4 (PMC6489261; doi:10.1186/s12967-019-1885-4)
Supplement: Supplementary file 1 — Additional file 1. Effects of I/R and RIPC on LDH release. [file 12967_2019_1885_MOESM1_ESM.pptx]

## Slide 1
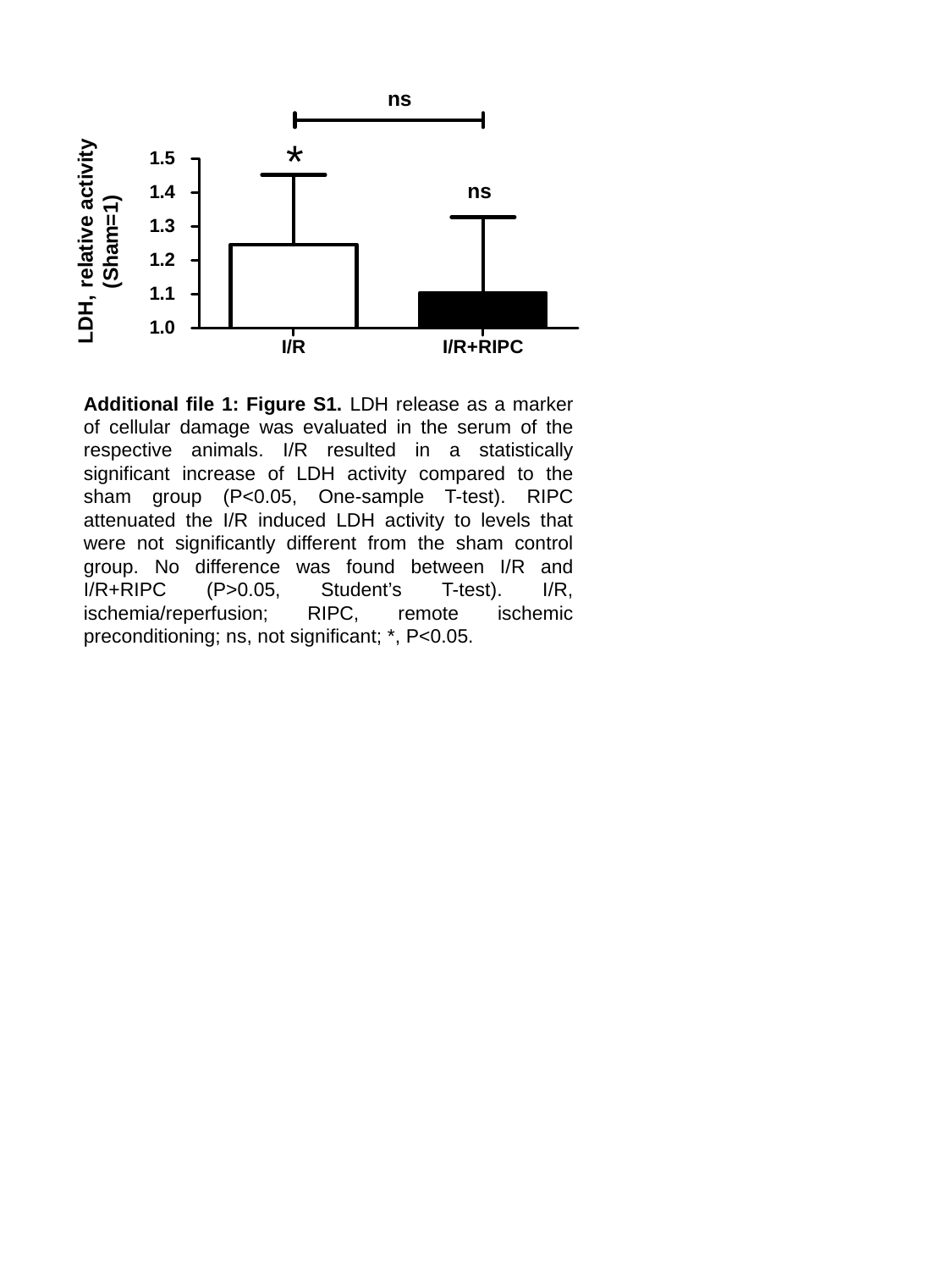

Additional file 1: Figure S1. LDH release as a marker of cellular damage was evaluated in the serum of the respective animals. I/R resulted in a statistically significant increase of LDH activity compared to the sham group (P<0.05, One-sample T-test). RIPC attenuated the I/R induced LDH activity to levels that were not significantly different from the sham control group. No difference was found between I/R and I/R+RIPC (P>0.05, Student’s T-test). I/R, ischemia/reperfusion; RIPC, remote ischemic preconditioning; ns, not significant; *, P<0.05.
